# Development of Antibiofilm Therapeutics Strategies to Overcome Antimicrobial Drug Resistance

**DOI:** 10.3390/microorganisms10020303

**Published:** 2022-01-27

**Authors:** Sahaya Nadar, Tabassum Khan, Simon G. Patching, Abdelwahab Omri

**Affiliations:** 1 Department of Pharmaceutical Chemistry, St. John Institute of Pharmacy and Research, Mumbai 400056, India; sahayan@sjipr.edu.in; 2 Department of Pharmaceutical Chemistry & Quality Assurance, SVKM’s Dr. Bhanuben Nanavati College of Pharmacy, Mumbai 400056, India; tabassum.khan@bncp.ac.in; 3 School of Biomedical Sciences and Astbury Centre for Structural Molecular Biology, University of Leeds, Leeds LS2 9JT, UK; 4 The Novel Drug & Vaccine Delivery Systems Facility, Department of Chemistry and Biochemistry, Laurentian University, Sudbury, ON P3E 2C6, Canada

**Keywords:** biofilms, infection, inhibitors, extracellular polymeric substances, antimicrobials, small molecules

## Abstract

A biofilm is a community of stable microorganisms encapsulated in an extracellular matrix produced by themselves. Many types of microorganisms that are found on living hosts or in the environment can form biofilms. These include pathogenic bacteria that can serve as a reservoir for persistent infections, and are culpable for leading to a broad spectrum of chronic illnesses and emergence of antibiotic resistance making them difficult to be treated. The absence of biofilm-targeting antibiotics in the drug discovery pipeline indicates an unmet opportunity for designing new biofilm inhibitors as antimicrobial agents using various strategies and targeting distinct stages of biofilm formation. The strategies available to control biofilm formation include targeting the enzymes and proteins specific to the microorganism and those involved in the adhesion pathways leading to formation of resistant biofilms. This review primarily focuses on the recent strategies and advances responsible for identifying a myriad of antibiofilm agents and their mechanism of biofilm inhibition, including extracellular polymeric substance synthesis inhibitors, adhesion inhibitors, quorum sensing inhibitors, efflux pump inhibitors, and cyclic diguanylate inhibitors. Furthermore, we present the structure–activity relationships (SAR) of these agents, including recently discovered biofilm inhibitors, nature-derived bioactive scaffolds, synthetic small molecules, antimicrobial peptides, bioactive compounds isolated from fungi, non-proteinogenic amino acids and antibiotics. We hope to fuel interest and focus research efforts on the development of agents targeting the uniquely complex, physical and chemical heterogeneous biofilms through a multipronged approach and combinatorial therapeutics for a more effective control and management of biofilms across diseases.

## 1. Introduction

The term biofilm refers to a community of stable microorganisms encapsulated in an extracellular matrix produced by themselves that develops in a multitude of biological and ecological environments [1,2]. Microbial biofilms are of grave health concern worldwide owing to their ability to be resistant to antibiotics, resist host immune response and combat extreme environmental stress, and their association with persistent infections [3]. Morbific microbes possess the ability to form biofilms in tissues and biomaterials, inducing chronic infections that are arduous to treat [4,5]. Common bacteria that form biofilms include *Pseudomonas aeruginosa* [6], *Staphylococcus epidermidis* [7], *Enterococcus faecalis* [8,9,10], *Staphylococcus aureus* [11,12,13,14], *Klebsiella pneumoniae* [15,16,17], *Streptococcus viridans* [18,19,20,21], *Escherichia coli* [22,23], and *Proteus mirabilis* [24,25]. Biofilm recalcitrance [26,27,28,29,30] is the potential of the microbes to sustain even in high concentrations of antibiotics, which leads to recurrence of infections and collapse of treatment. The microbes enclosed within the biofilm have proven to be more resistant towards classic antibiotic therapy in contrast to the planktonic cell population. This recalcitrance is revocable by disrupting the biofilm and reinstating the microbes to the planktonic state [31]. Patients with ingrained medical devices such as prosthetic heart valves, catheters, joint prosthesis, cardiac pacemakers, dental implants and contact lenses have utmost risk of biofilm-based nosocomial infections [32,33,34]. Such foreign bodies provide an exemplary surface for the adhesion of bacterial cells, which can be facilitated by non-specific factors such as shear forces, hydrophobicity and electrostatic interactions [35,36,37].

A common example of biofilms covering abiotic surfaces is their formation on dental restorative and implant materials, where oral bacteria adhere to hydrophobic and hydrophilic abiotic surfaces and biofilms accumulate more readily on rough than on smooth surfaces [38,39]. This can result in periimplantitis, a destructive inflammatory process that affects the soft and hard tissues surrounding dental implants and may result in implant failure. There is a multitude of methods for decontaminating pathogenic microorganisms on dental implants [40].

Exposure of bacteria to concentrations of an antibiotic that are lower than the minimum inhibitory concentration (MIC), known as subinhibitory concentrations, can enforce a higher capacity for biofilm formation, which in turn can result in a decreased susceptibility to antibiotics [41,42,43,44]. For example, subinhibitory antibiotic concentrations have recently been shown to enhance biofilm formation of clinical *Enterococcus faecalis* isolates [45]. The subinhibitory concentration may be introduced by improper dosing of an antibiotic or could be created in difficult to reach local regions such as in a dental root canal. The inability of antibiotic treatments to eliminate bacterial biofilms at subinhibitory concentrations has hastened the quest for new antibiofilm agents and strategies. In this review we therefore look at how biofilms are formed and how this process could be inhibited, then consider possible antibiofilm agents and strategies in the quest to overcome antimicrobial drug resistance.

## 2. Biofilm Formation

Biofilm biogenesis is a dynamic process that involves a consecutive series of steps [46,47]. The process of biofilm formation commences by the bacteria approaching to a surface. Most bacteria have the ability to switch between two forms that are planktonic single cells and sessile biofilms. The planktonic cells and biofilms vary remarkably in their gene expression, morphological and physiological facets. The sessile cells are encapsulated by extracellular polysaccharides (EPS) and demonstrate increased production of surface adherents, innate tolerance to antibiotics, and soaring resistance towards environmental stress.

**Figure 1:**
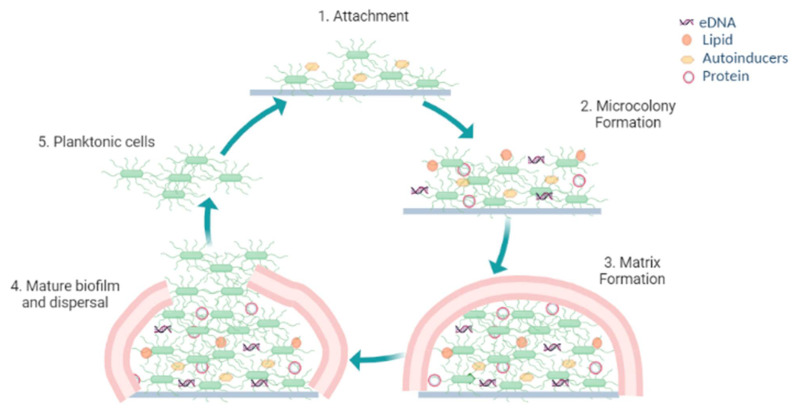
Biofilm formation process.

The biofilm formation process (Figure 1) involves different phases:

### 2.1. Reversible Attachment

Under favorable conditions, a single planktonic cell migrates and reversibly attaches itself onto a surface initiating the first phase of the biofilm biogenesis process [48,49]. Being reversible, this attachment involves weak interactions such as electrostatic, van der Waals or hydrophobic interactions. Cell appendages such as pilli, flagella or fimbriae provide robustness and adhesion to the surface of attachment. On attachment the cells become further become encapsulated in EPS [50,51].

### 2.2. Microcolony Formation with Quasi-Irreversible Adhesion

In this stage the planktonic cells become prominently more layered and form a systematic microcolony along with water channels making it an irreversible adhesion. Colonization, a hallmark feature of biofilms, plays a vital role in its dormancy and virulence. Once the cells securely adhere to an appropriate surface countless microbes pile up and secrete EPS that acts as a sealant to fix the microorganisms. After these synchronized steps the microcolonies are formed [52,53,54].

### 2.3. Biofilm Matrix Formation

The EPS produced by the adherent forms a matrix within which the cells build their community and attain their maximum cell density. The EPS encapsulating the cells in a biofilm is an amalgamation of constituents, including extracellular-DNA (e-DNA), autosensing molecules, persister cells, proteins, lipids and polysaccharides [55,56,57,58]. The polysaccharides in the matrix provide strength to the cells within the biofilm, such as adherence, shielding, and structural rigidity [59,60]. Colonization is facilitated by the aggregative polysaccharides that act as glue and also provide protection from physical stresses inflicted by the moving fluid depriving the cells of nutrients [61,62,63]. Organisms inherit different kinds of polysaccharides that exhibit key roles in biofilm integrity (Table 1). The nucleic acids like e-DNA, extracellular-RNA (e-RNA) and ribosomal DNA interact with different EPS granting nutrients, providing structural stratification and protection against any gene transfer to the biofilm. The proteins present in the matrix bestow structure and stability to the biofilm [64,65,66]. The persister cells contribute to a small community of dormant cells that display utmost resistance towards antimicrobials [67,68,69,70].

### 2.4. Maturation of the Biofilm and Detachment

With availability of favorable conditions and nutrients the cells grow and differentiate to form mature biofilms which have a spatial architecture. This well developed biofilm resembles a communal group arbitrated by chemical signaling molecules liberated by bacterial inhabitants within the biofilm. On maturation, the microcolonies of cells release individual planktonic cells now capable of travelling to a new surface, thus leading to outspread of the bacterial infestation [98,99,100,101].

## 3. Mode of Action of Antibiofilm Agents

### 3.1. Bacterial Surface Attachment Inhibition

Bacterial appendages like flagella or fimbriae aid their attachment to surfaces, so inhibition of these appendages can be an approach to avert adhesion. Surface coating or surface modification with agents having antibacterial properties is an emerging technique to hostile microbial adhesion and proliferation [102,103,104,105,106]. Inhibition of bacterial adhesion can be achieved by surface coating with biocidal agents or specific polymers having an ability to inhibit the cells impending the surface. Indeed, novel polysaccharides from Antarctic sponge-associated bacteria and lake macroalgae have recently been used to hinder the adhesion of bacteria [107,108]. Surface topography using nanotechnology has recently been explored to generate antibacterial surfaces [109,110,111,112].

### 3.2. Interfering with Quorum-Sensing

**Figure 2:**
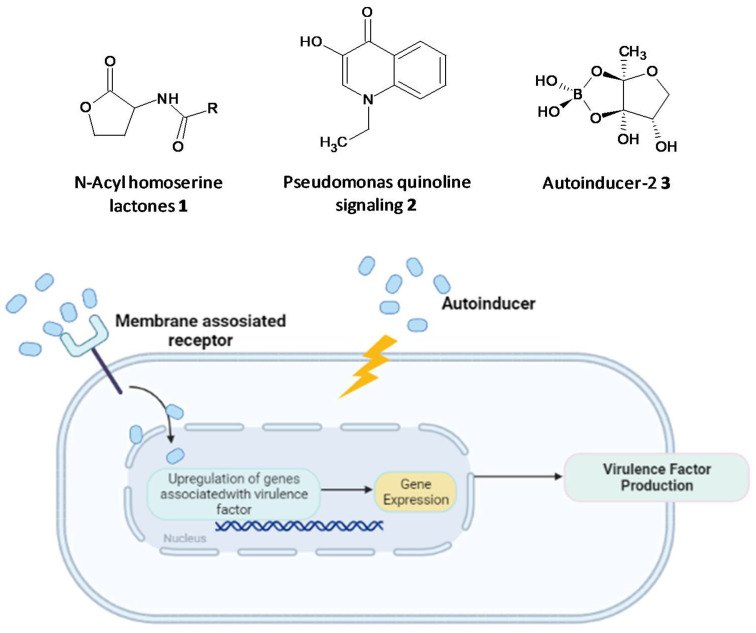
Chemical structures of some autoinducers (**top**) and production of virulence factors (**bottom**).

Microbial cell-to-cell communication at the molecular level, which enables the microbes to reciprocate to surrounding changes, is permitted by a mechanism called quorum-sensing (QS). QS is reliant on the binding of an autoinducer to an analogous gene regulator, which activates the ensuing transcriptions [113,114,115,116,117]. Examples of autoinducers are N-acyl homo-serine lactones (AHLs) (**1**) and Pseudomonas quinoline signaling molecules (**2**) present in Gram-negative species, short peptide signals present in Gram-positive species, and autoinducer-2 (**3**) molecules found in both species (Figure 2). These help to control population density, swarming motility, virulence and biofilm formation [118,119,120]. The autoinducers initiate the formation of virulence factors that aid invasion and persistence in a vulnerable host. QS systems are associated with upregulation of gene expression through the accessory gene regulator (*agr*) system, forming virulence factors such as adhesins, toxins, hemolysin and autolysins in Gram-positive staphylococcal infections or siderophores, exoproteases, rhamnolipids and exotoxins in *P. aeruginosa* [121,122,123,124]. The formation of biofilms and related virulence factors by infective microbes requires cell-cell communication, hence agents acting as QS inhibitors and specifically targeting the AHL-QS system in bacteria have been widely explored for their efficacy using in vitro and in vivo models [125,126,127].

### 3.3. Nucleotide Second Messenger Signaling Modulating Molecules

The second messenger cyclic di-guanosine monophosphate (c-di-GMP) has emerged as a signaling molecule in Gram-positive and Gram-negative bacteria governing the process of biofilm formation, biosynthesis of EPS, virulence and suppression of cell motility. The enzyme diguanylate cyclase is essential for synthesis of c-di-GMP, inhibition of which has proven to terminate biofilm formation, alluding to the significance of c-di-GMP in the bacterial signaling process [128,129,130,131,132,133,134,135].

### 3.4. Bacterial Genetic Biodiversification Inhibitors

Genetic biodiversification in bacteria leads to emergence of newer subpopulations, which have been ascribed to be resistant to antibiotic therapies and environmental stressors. Horizontal gene transfer with conjugation plays a crucial role in the outspread of resistance in biofilm colonies [136,137,138]. The social evolution theory anticipates that inhibiting shared traits among the subpopulations could be a viable solution for eradicating the biofilm. The fact that the organism in a subpopulation relies on a shared EPS makes it an interesting target to combat genetic diversification. The spatial structure and heterogeneity provided by biofilms lead to increased genetic diversity [139].

### 3.5. Biofilm Dispersal Inducers

Biofilm dispersal is initiated by the disruption of the EPS matrix to release the microcolonies of planktonic cells that migrate and adhere to new surfaces. Antibiofilm agents that can abet the process of dismantling the biofilm has provided research strategies for designing new biofilm dispersal inducers [140,141,142]. This dispersal process provides an opportunity to target the microorganisms since they now exist in their viable form, a way more susceptible form permitting the attack of standard antimicrobials comparable to cells residing in the biofilm [143,144]. Biofilm dispersal agents have triggered the interest of researchers to design combination treatments along with antibiotics.

## 4. Bacterial Resistance towards Antibiotics

When antibiotics are intended to treat biofilm infestation, they must have the ability to cross the biofilm matrixome to target the cells embedded within. Although this is not the case most of the time as the antibiotics fail to cross the biofilm extracellular matrix due to surface modification of the biofilm causing decreased influx. The mechanisms by which antibiotic resistance develops are a crucial determinant factor in the survival of biofilm microbes. The microbes that form biofilms inherently undergo high mutation that allows them to evolve resistant mechanisms providing fortuity for genes to develop enzymes that inactivates the antibiotics or extrudes the antibiotics by efflux pumps [145,146,147,148].

**Figure 3:**
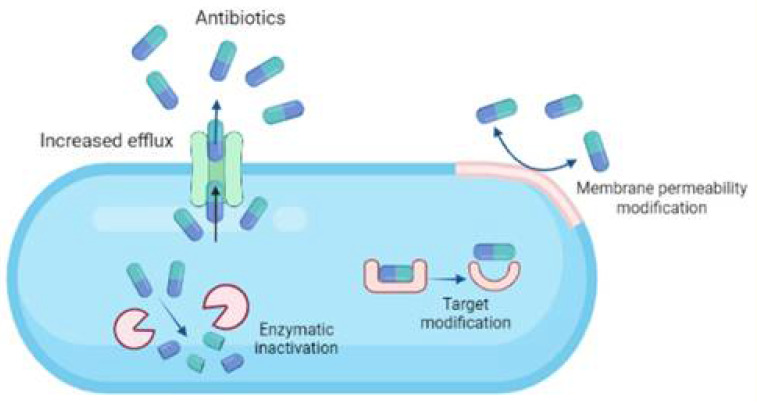
Major mechanisms involved in the development of antibiotic resistance among bacterial species.

Four major mechanisms are involved in the antibiotic resistance developed among bacterial species (Figure 3):

i. Modifying cell permeability to restrict the influx of antibiotics into the cells.
ii. Altering the cellular targets to which the antibiotics bind, rendering them inactive.
iii. Enzymatic cleavage of the antibiotics making them ineffective.
iv. Upregulation of efflux pumps to expel the antibiotics out of the cellular membrane.

Horizontal gene transfer in plasmids and the portability of human carriers has resulted in proclamation of drug resistance over a capacious microbial subdivision and microenvironment [149,150,151,152,153].

## 5. Emerging Antibiofilm Agents

Biofilms embrace the capability to resist and survive harsh environmental conditions and defeat the host immune system, so there is a desire for exploring new antibiofilm agents. Emerging biofilm control measures such as small molecule inhibitors, quorum quenching agents, antimicrobial peptides (AMPs), efflux pump inhibitors (EPIs), quaternary ammonium compounds (QACs), and natural phytoconstituents are gaining acclaim to selectively act by different mechanisms and combat the resistance. With the unfolding knowledge of biofilm biogenesis and microenvironments, various agents and newer emerging technologies have provided novel approaches for selectively targeting the biofilm by annihilating the biofilm or suppressing its formation.

### 5.1. Inhibition of Persister Cell Formation by a Synthetic Diterpene

**Figure 4:**
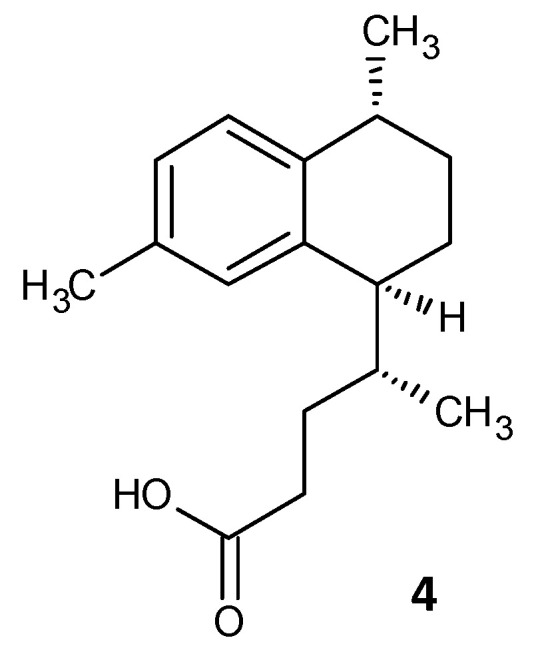
Chemical structure of DMNP.

Tkachenko et al. identified a synthetic diterpene derivative as a lead molecule proficient in repressing resistance and annihilating the biofilm formation in *Mycobacterium smegmatis*. Persister cell formation in Mycobacterium is highly dependent on the alarmone (p)ppGpp and its essential Rel protein. The analogue 4-(4,7-di-methyl-1,2,3,4-tetrahydro-naphthalene-1-yl)pentanoic acid (DMNP) (**4**) (Figure 4) was found to inhibit RelMsm activity of (p)ppGpp-synthesis in a concentration-dependent manner [154]. Furthermore, docking studies suggested the interaction of DMNP with RelZ and RelMsm proteins and high affinity with their GTP binding sites, consequently impeding their (p)ppGpp-synthesizing activity [155].

### 5.2. Inhibition of Sortase A by 1,2,4-Oxadiazole Topsentin Analogs

**Figure 5:**
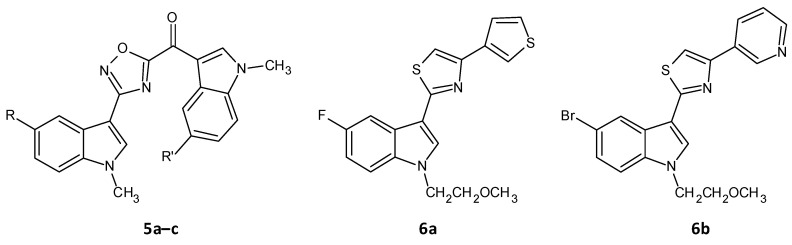
Chemical structures of potent 1,2,4-oxadiazole topsentin analogs.

A series of seventeen 1,2,4-oxadiazole topsentin analogs was synthesized by Parrino et al. and assessed for their biofilm inhibition, by targeting the membrane enzyme transpeptidase sortase A (SrtA), which attaches surface adhesive molecules to the cell wall in Gram-positive organisms. All these compounds inhibited biofilm formation in *S. aureus* species with BIC50 values less than 10 μM for the most potent derivatives (**5a**–**c**). The potent analogues displayed BIC50 values for *S. aureus* in the range of 0.7 to 9.7 μM, and additionally showed a superior enzyme inhibition with IC50 values of 2.2 to 10.4 μM. SAR analysis revealed the significance of the presence of the -N=C-O- group in the oxadiazoles for dual antibacterial and biofilm inhibitory activity [156]. In a similar work by Carbone et al., thiazole analogs of nortopsentin were synthesized of which the potent derivatives **6a**–**b** showed BIC50 values against *S. aureus* of 3.9 and 1.0 µM (Figure 5 and Table 2) [157].

### 5.3. Amide Chalcones

**Figure 6:**
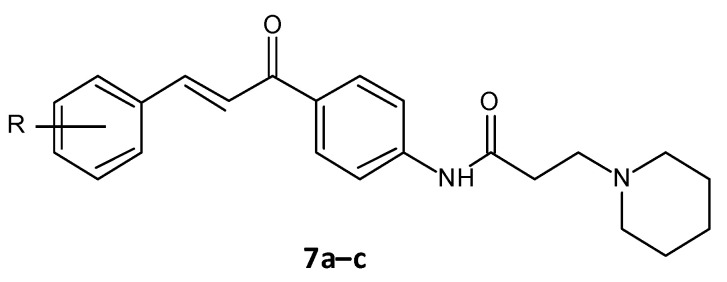
Chemical structure of amide chalcones.

El-Messerya et al. synthesised a panel of amide chalcones linked with different secondary amines and assessed them for in vitro antibacterial activity and their antibiofilm activity. A minimum bactericidal concentration (MBC) value of 2.0 mg/mL against *S. aureus* equivalent to the standard ampicillin was shown by compound **7a**. Compounds **7a**, **7b**, and **7c** (Figure 6) displayed a significant biofilm inhibition with IC50 values in the range of 2.4 to 8.6 mg/mL against *S. aureus, Micrococcus luteus* and *P. aeuroginosa* (Table 3) [158,159].

### 5.4. Cajaninstilbene Acid Derivatives

**Figure 7:**
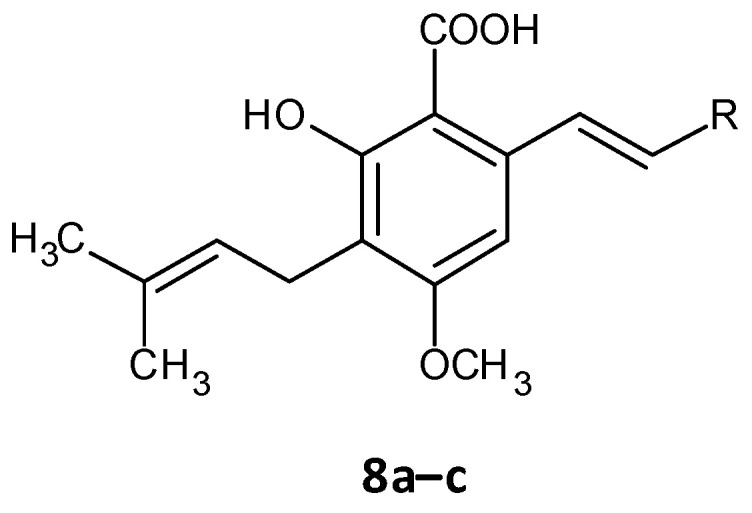
Chemical structure of cajaninstilbene acid derivatives.

Chen and co-workers recently developed cajaninstilbene acid derivatives and evaluated their ability to inhibit biofilm formation. Of the synthesized analogues, compounds **8a**, **8b** and **8c** (Figure 7) exhibited promising antibiofilm activity, furthermore **8c** displayed potent biofilm inhibition with a ratio of 49.50 ± 1.35% at 50 μM (Table 4). Additionally, compound **8c** showed suppression on expression of lasB-lacZ and pqsA-lacZ involved in the QS network pathway in *P. aeruginosa*. Thereby proving compound **8c** as a promising lead with inhibition of QS and associated biofilm formation in *P. aeruginosa* [160,161].

### 5.5. Quorum Quenching Agents

**Figure 8:**
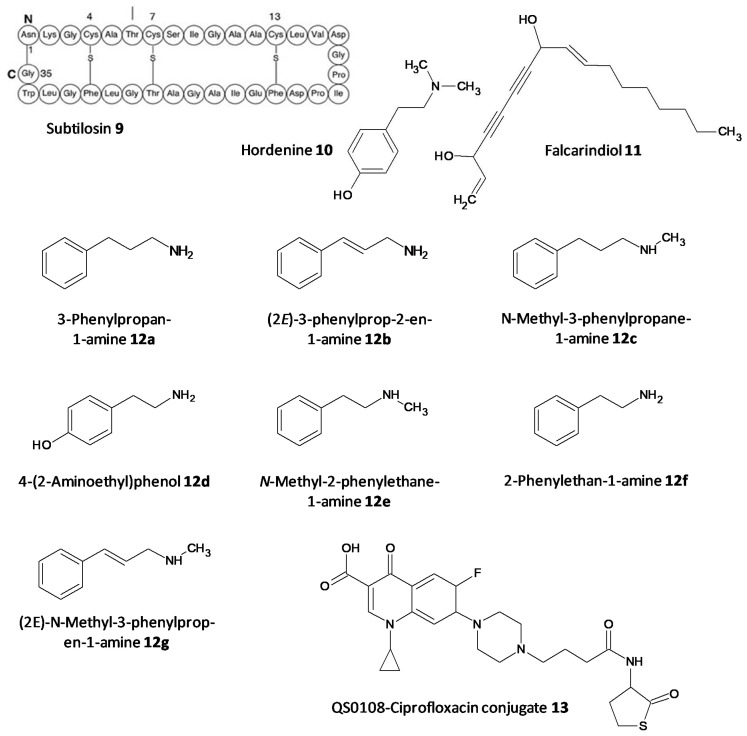
Chemical structures of various quorum sensing inhibitors.

The persister cells have the ability to communicate amongst themselves leading to virulence and the generation of resistance. Different quorum quenching agents have been explored in order to find adjuvant therapy. The bacterial QS inhibitory effect of subtilosin (**9**), a cyclic lantibiotic formed by *B. subtilis* KATMIRA1933, was assessed by Algburi et al. [162]. Subtilosin shows its effect by targeting the surface receptor and binding to the bacterial cell membrane by electrostatic forces. The study revealed that at a concentration of 15.1 μg/mL an inhibition of 80% of *L. monocytogenes* and about 60% of *E. coli* biofilms was seen. Moreover, subtilosin decreased the autoinducer-2 formation in *Gardnerella vaginalis* at a concentration of 3–4 μg/mL [152]. Zhou et al. evaluated the QS inhibitory potential of hordenine (**10**) isolated from sprouting barley towards *P. aeruginosa.* It was found to inhibit the autoinducer AHLs at concentrations of 0.5 to 1.0 mg/ mL. Additionally, it also remarkably suppressed the QS associated genes *lasR*, *rhlR*, *rhlI* and *lasI* [163]. In another work by Zhao et al. the QS inhibitory effect of falcarindiol (**11**) against *P. aeruginosa* infestation was assessed. Biofilm formation and associated virulance factors were significantly inhibited at subinhibitory concentrations. Also, there was appreciable downregulation of the mRNA expression of QS associated genes *lasI, lasB*, *rhlA*, *pqsA*, *rhlR, phzH* and *rhl I* [164]. QS and biofilm inhibitory effects of a few hordenine derivatives towards *P.* aeruginosa and *Serratia marcescens* was recently analysed by Liu et al. Derivatives **12a**–**g** exhibited superior QS inhibitory activity and biofilm inhibition towards *P.* aeruginosa. Additionally, analogues **12a**–**c** and **12g** displayed remarkable QS inhibition against *S.* marcescens. SAR studies revealed essential factors involved in activity like alkyl chain length, presence or absence of amino or hydroxyl groups and unsaturation in the aromatic benzene ring [165]. A thiolactone analog of AHL covalently linked to ciprofloxacin (QS0108) (**13**) was developed by Ganguly et al. to assess them as inhibitors of AHL-2 in *P. aeruginosa.* This system effectively disrupted dormant and mature biofilms compared to antibiotic treatment alone (Figure 8) [166].

### 5.6. Antimicrobial Peptides

AMPs are emerging as attractive antibiofilm agents owing to various properties that they display, such as a broad-spectrum of antimicrobial activity, decreased resistance and synergistic effects shown with few antibiotics. Indeed, these properties mean that AMPs could become the next generation of antimicrobials to curb the biofilm related resistance shown by current antibiotics.

Heinonen et al. recently explored the antibiofilm effect of TAT-RasGAP317-326, an AMP made of a TAT HIV 48–57 sequence which gives it cell permeability, and a sequence of ten amino acids obtained from the Src homology domain of p120 RasGAP on biofilms of *P. aeruginosa, A. baumannii* and *S. aureus.* It was observed that TAT-RasGAP317-326 attenuated biofilm formation at concentrations similar to or twice the MIC value obtained for planktonic cells. Additionally, TAT-RasGAP317-326 curbed the growth and spread of *P. aeruginosa* and *A. baumannii* preformed biofilms at twice the concentration. This study proclaims TAT-RasGAP317-326 as a propitious antibiofilm AMP [167].

Wuersching et al. assessed the effect of AMPs LL-37 (also known as cathelicidin) and human lactoferricin (LfcinH) on the growth of planktonic cells and biofilm formation in anaerobes associated with oral pathogenesis. Suspensions of multi-species of facultative anaerobic bacteria (FAB) including *Actinomyces naeslundii, Streptococcus mutans* and *Streptococcus sanguinis* or obligate anaerobic bacteria (OAB) including *Parvimonas micra*, *Veillonella parvula* and *Fusobacterium nucleatum* were subjected to concentration ranges of LL-37 and LfcinH. Compared to LfcinH, prominent inhibitory threshold concentrations of LL-37 were noticed (*p* < 0.0001) but the biofilm mass was also decreased better by LL-37 compared to LfcinH, highlighting the scope of LL-37 as a better AMP [168,169].

Ciandrini et al. investigated the synergistic action of AMPs citropin 1.1, temporin A, Pal-KGK-NH_2_ and CA(1–7)M(2–9)NH_2_ towards methicillin-resistant *S.s aureus* (MRSA) biofilms formed on a polystyrene surface and a central venous catheter. The combination evidently inhibited biofilm formation, although, disruption of preformed biofilms was tedious and achieved after 24 h of contact [170,171].

Festa et al. recently worked on the AMP 1018-K6 as an antibiofilm agent against MRSA and enterotoxigenic *S. aureus* isolated from cheese. This peptide exhibited remarkable eradication of preformed Staphylococcal biofilms within 15 min. Moreover, it prevented further formation of biofilms and displayed bactericidal action against the planktonic cells [172]. In a continuation of the work on 1018-K6, Colagiorgi et al. worked on the antibiofilm ability of the food pathogen *Salmonella enterica.* Of the 42 strains included in the study, 1018-K6 profoundly decreased the biofilm formation in several *S. enterica* strains at subinhibitory concentrations [173].

### 5.7. Antibiotics Affecting Bacterial Cell Permeability

Antibiotics affecting cell permeability target several components of the cell wall and its synthesis. The peptidoglycan class of antibiotic vancomycin inhibits the cell wall synthesis by complex formation with the D-Ala-D-Ala subunit at the carboxyl terminal in a peptidoglycan chain [174,175,176]. AMPs like polymyxin B pile up in the outer membrane by binding to lipid A and eventually invade the inner membrane making its way into the cytoplasm. The resistance to this antibiotic is seen by either mutations in the governing systems PmrAB and PhoP/PhoQ or modification of the phosphate functional groups of lipid A [177,178].

### 5.8. Enzymatic Cleavage Inhibitors

**Figure 9:**
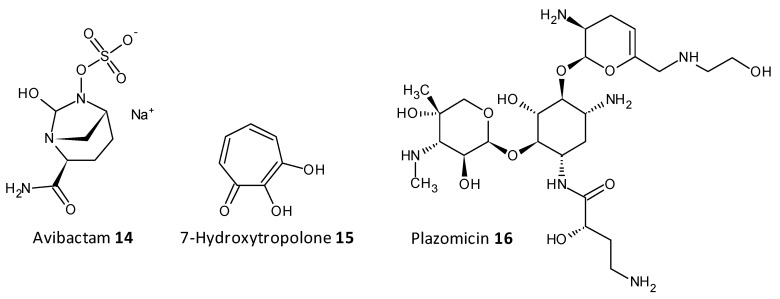
Chemical structures of some enzymatic cleavage inhibitors.

On being exposed to an antibiotic the bacterial cells inturn release enzymes which act in a defensive way in the extracellular space, cleaving and deactivating the antibiotic. The resistance to beta-lactam antibiotics has been associated to the enzyme beta-lactamase. A combination of ceftazidime and a beta-lactamase inhibitor avibactam has been explored for its antibiofilm activity towards carbapenemase-producing *K. pneumoniae*. Avibactum (**14**) was also proven to irreversibly curb the β-lactamase enzyme from *Mycobacterium tuberculosis* [179,180,181,182]. 7-Hydroxytropolone (**15**) acts as an inhibitor of the enzyme aminoglycoside-2”-O-adenylyltransferase and was active against bacterial strains resistant to amioglycosides. The structure of 7-hydroxytropolone, exhibits an eccentric vicinal positioning of the oxygens which aids in the enzyme inhibition [183]. Plazomicin (ACHN-490) (**16**) a neamine derivative was designed by modifying the sites that displayed affinity to the resistance caused by aminoglycoside-modifying enzymes allowing it to preserve the antimicrobial activity towards pathogens that possess aminoglycoside resistance genes [184,185] (Figure 9).

### 5.9. Efflux Pump Inhibitors

**Figure 10:**
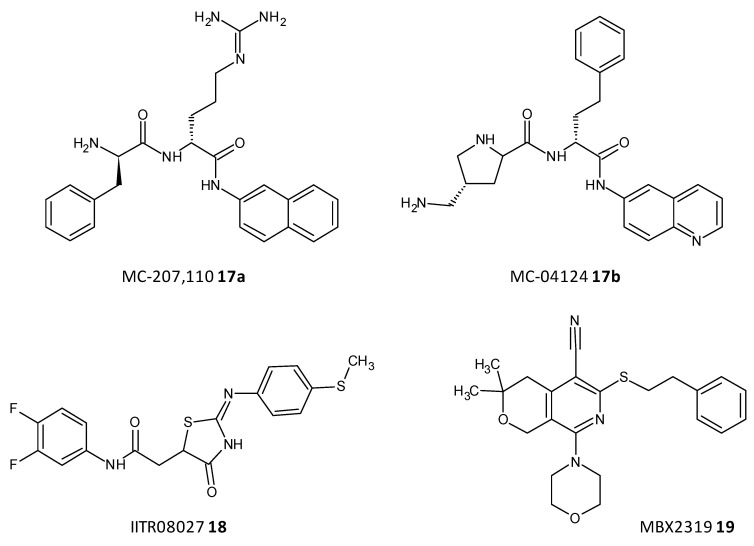
Chemical structures of some efflux pumps inhibitors.

Efflux of antibiotics, by overexpression of efflux pumps, leads to their extrusion out of the cell, making them inactive. EPIs are a recent class of compounds which specifically aim to prevent the efflux of antibiotics out of the cell, which is a determinant of the resistance shown toward antibiotics. A peptidomimetic efflux pump inhibitor MC-207,110 (**17a**), also known as phenylalanyl arginyl β-naphthylamide, and its analogue MC-04124 (**17b**) (Figure 10) enhanced the antimicrobial activity of erythromycin and levofloxacin against clinical strains of *P. aeruginosa* overexpressing MexAB-OprM [186,187]. A synthetic small molecule IITR08027 (**18**) (Figure 10) showed reversal of resistance towards fluoroquinolones in clinical strains of *A. baumannii* overexpressing multidrug and toxic compound extrusion (MATE) efflux pumps and the recombinant strains of *E. coli*. IITR08027 disrupts the proton gradient important for activating the efflux pump [188]. MBX2319 (**19**) a pyrazolopyridine analogue displayed inhibition of AcrAB-TolC-overexpressing in *E. coli* and potentiated the efficacy of antibiotics like levofloxacin, ciprofloxacin, and piperacillin [189] (Figure 10).

### 5.10. Quaternary Ammonium Compounds

QACs represent a class of broad-spectrum antimicrobials possessing a central amphiphilic core and a lipophilic alkyl side chain linked to a hydrophilic quaternary ammonium framework. Although QACs imitate the action of the AMPs they have a comparatively simplified structure. The mode of action of these agents as antimicrobials is by cleavage of the cell membrane, which consequently leads to nutrient leakage followed by cell lysis and death. Although, this class of compounds have already been established as disinfectants, antiseptics and preservatives their role as antibiofilm agents has only recently been recognised.

In a study by Kumar Tiwari et al. two quaternary ammonium methacrylate (QAM) derivatives, dimethylaminododecyl methacrylate (DMADDM) (**20**) and dimethylaminohexadecyl methacrylate (DMAHDM) (**21**), were designed and evaluated for their efficacy as antibiofilm agents against *E. faecalis, Streptococcus gordonii, Actinomyces naeslundii,* and *Lactobacillus acidophilus* using chlorhexidine and sodium hypochlorite as standards. In particular, the minimal biofilm inhibitory concentration (MBIC) for DMADDM and DMAHDM against a combination of four endodontic bacteria was 25 μg/mL and 6.25 μg/mL, respectively [190]. Daood et al. investigated quaternary ammonium silanes (QASs) (**22**) as biofilm disruptors by exposing them to *S. mutans* and *L. acidophilus* preformed biofilms over dentine disks, at different concentrations. Inhibition of enzyme SrtA, responsible for the adhesion of proteins onto the cell membrane and connecting the proteins to form pili, was studied at a concentration of 2% QAS dilution and exhibited significant reduction with an IC50 value of 3.3 ± 2.7 μM, a more potent value as compared to polyhexamethylene biguanide taken as positive control, IC50 = 24.5 ± 4.1 μM [191].

**Figure 11:**
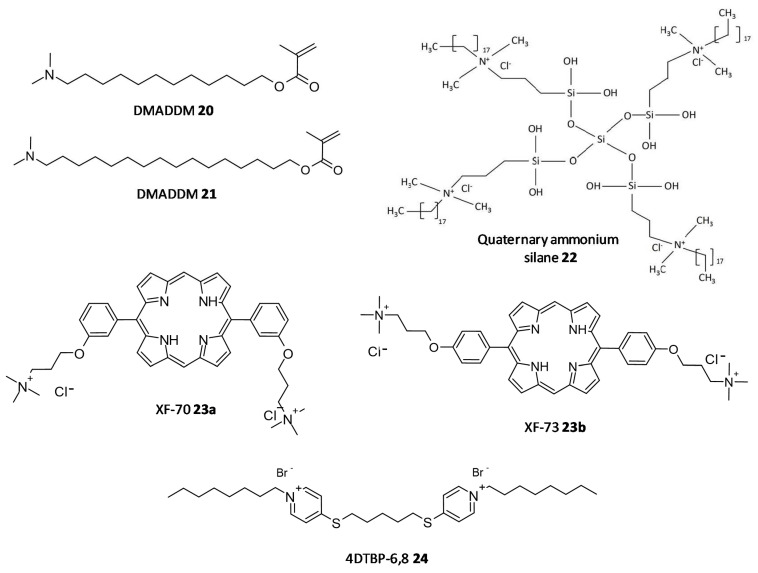
Chemical structures of some quaternary ammonium compounds.

In a study conducted by Ooi et al., two distinct QACs having a dicationic porphyrin core, XF-70 (**23a**) and XF-73 (**23b**), were evaluated for their disruption of *S. aureus* biofilms. Both analogues entirely disrupted preformed *S. aureus* biofilms at a concentration of 2.6 μM [192,193]. The analogue XF-73 is currently being developed as a topical preparation by Destiny Pharma (Brighton, UK) and has started with a phase-II trial to assess its effect on patients with post surgical Staphylococcal nasal infestation. A phase–I trial of XF-73 has shown noteworthy positive results [194]. Murakami et al. evaluated the effectiveness of 4,4′-(α,ω-hexamethylenedithio) bis (1-octylpyridinium bromide) (4DTBP-6,8) commonly known as gemini QACs a seventh generation BisQACs as an antibacterial and antibiofilm agent towards *P. aeruginosa*. A susceptibility assay for biofilm cells was performed which showed the numbers of surviving cells with cetylpyridinium chloride and benzalkonium chloride used as reference was 984- and 186- fold higher compared to cells treated with 4DTBP-6,8 (**24**) (Figure 11) [195].

### 5.11. Natural Compounds

Dong et al. evaluated resveratrol as a biofilm inhibitor against *Aeromonas hydrophila*. The significant reduction in biofilm formation using resveratrol (**25**) was noted at concentrations higher than 0.25 μg/mL and a 49.11% reduction in biofilm biogenesis was observed with 4 μg/mL [196]. Oh et al. investigated Raffinose (**26**), which is a α-galactosyl derivatives of sucrose, and their isolate from ginger extract and evaluated for their ability to prevent biofouling in membrane bioreactors involved in membrane filtration and treatment of waste water. It was observed that the extract significantly reduced 25–52% of the *P. aeruginosa* and *S. aureus* co-culture biofilms at a concentration range of 0–1000 μM. In addition, Raffinose also decreased the transmembrane pressure in lab-scale membrane bioreactors compared to furanone C-30 used as control [197].

In a study by Husain et al., Pseudomonas species producing metallo-b-lactamase (MbLs) enzymes from camel meat were isolated and evaluated for their ability to form biofilms. Additionally, the effect of the flavone naringin (**27**) on the biogenesis of biofilm against the isolated Pseudomonas species was assessed by in silico and in vitro studies. A total of 55% isolates were found to produce MbLs. Naringin attenuated up to 57% of the biofilm formation in the isolated Pseudomonas species. Naringin remarkably turned down biofilms EPS and alginate density. Disruption of preformed biofilms from 32–60% was seen at respective 0.50 MICs. Naringin can thus be explored as a promising food preservative against foodborne Pseudomonas species forming biofilms [198].

In recent work by Lyu et al. the biofilm inhibition ability of ursolic acid (**28**) was evaluated towards oral Streptococci species. Ursolic acid being a natural product can be derived from plant parts such as privet leaves, berries, loquat leaf, paulownia leaves and iron holly. It was observed that ursolic acid inhibits multi-species biofilms of *S. mutans, S. gordonii* and *S. sanguinis* at a concentration of 7.80 μg/mL. Moreover they inhibited specifically glucosyltransferases the prime cariogenic component of oral biofilms and displayed relatively less cytotoxicity towards human oral cells [199]. Wei et al. and Pun et al. recently investigated the outcome of phloretin (**29**) on *Listeria monocytogenes* biofilm formation. Phloretin in sub-MIC levels was used at different temperatures of 37 °C and 4 °C to treat the biofilm. It showed maximum inhibition of the biofilm up to 60% with a concentration of 20 μg/mL. Moreover, the amount of biofilm aggregation and adhesion in *L. monocytogenes* was subsequently diminished. The thickness of the biofilm was lessened by 2 μm at a concentration of 20 μg/mL. The mode of biofilm inhibition study revealed the role of phloretin reducing the QS related gene *agr* by 50% with 20 μg/mL phloretin [200,201].

Wang et al. investigated Baicalin (**30**), a natural compound derived from the roots of *Scutellaria baicalensis*, and evaluated its ability to inhibit biofilm formation in *Staphylococcus saprophyticus* and its QS by selectively inhibiting the MsrA drug efflux pump. The study displayed promising results with baicalin decreasing biofilm biogenesis, and bacterial aggregation by downstreaming the mRNA transcription proportion of the QS regulators agrA, agrC, sarA and RNAIII [202,203].

**Figure 12:**
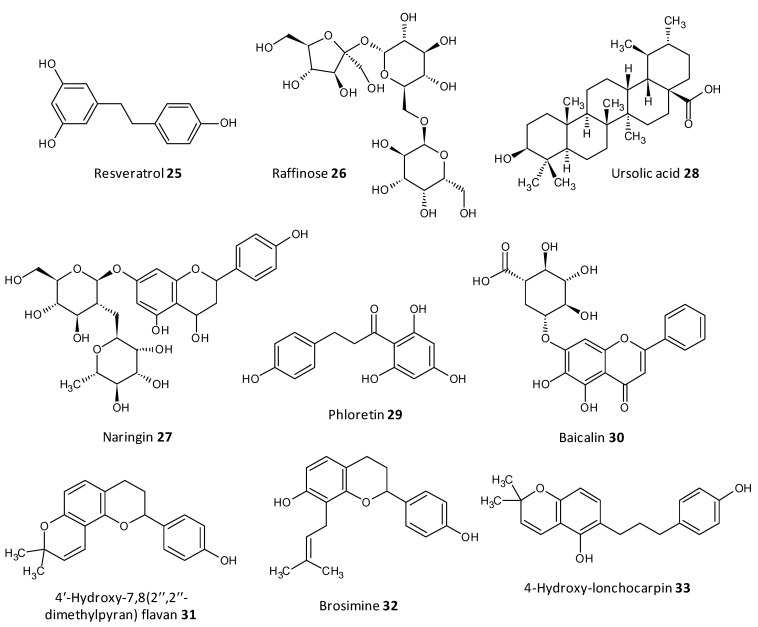
Chemical structures of some natural antibiofilm agents.

Reis et al. evaluated three flavonoids from *Brosimum acutifolium*, 4′-hydroxy-7,8(2″,2″-dimethylpyran) flavan (**31**), brosimine b (**32**) and 4-hydroxy-lonchocarpin (**33**), as antibiofilm agents. A and B decreased the viability in preformed *S. aureus* biofilms upto 73% at a concentration of 50 μM. Additionally, B decreased the biofilm biomass upto 48% at a concentration of 100 μM whereas C was unable to reduce the biofilm biomass. B at a concentration of 100 μM curbed 70–98% of planktonic cells in a 24-old MRSA biofilm (Figure 12) [204].

## 6. Investigative Strategies to Eradicate Biofilms

**Figure 13:**
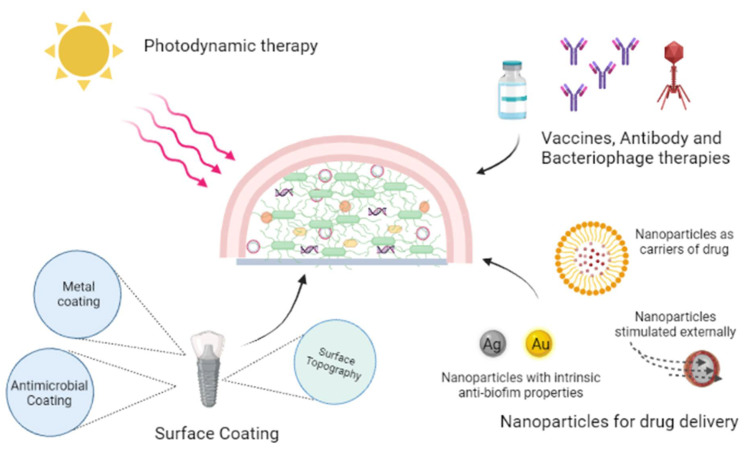
Investigative strategies to eradicate biofilms.

The emergence of resistance to existing clinical antimicrobials and the high dose regime required to curb biofilm associated infection conditions, and the paucity of a consummate antibiofilm therapy necessitates the need for novel strategies to thoroughly eradicate biofilms. Some burgeoning strategies are highlighted in this review (Figure 13).

### 6.1. Photodynamic Therapy

Photodynamic therapy (PDT) has prospective use in elimination of biofilm infections associated with surface wounds. In PDT mild visible light of specific wavelength is used along with innocuous photosensitizers to form cytotoxic reactive oxygen species (ROS) thus killing the bacteria. In a study conducted by Ronqui et al., synergistic therapy of PDT followed by ciprofloxacin treatment exhibited a remarkable reduction of biofilms, with a 5.4 and 7 log reduction for *S. aureus* and *E. coli* biofilms, respectively. Hypericin-loaded nanoparticles combined with PDT has been investigated, the study showed that PDT treatment offered quick wound healing and formation of collagen fibers in rats. In PDT appropriate care should be taken to avoid exposure of a patient’s eyes to the laser light [205,206,207,208].

### 6.2. Antibodies and Macrophages

Antibodies targeted towards bacterial surface attachment, if used prophylactically, can prevent biofilm biogenesis [209,210]. A human monoclonal antibody mAb 3H3, was assessed by Tursi et al. for its pan-amyloid binding ability. The antibody disrupted biofilms formed by *S.* *typhimurium* and eventually enhanced the efficacy of antibiotic treatment *in vivo*. In mice, a 3H3 injection showed antibiotic conciliated clearance of *S. typhimurium* biofilms in catheters [211,212,213,214]. Sun et al. used a mixture of two MAbs, 12C6/12A1 and 3C1/12A1, which synergistically reduced the attachment and accumulation of *S. epidermidis* up to 87% [215]. A cocktail of macrophages has the ability to avert *Proteus mirabilis* biofilms formed on catheters [216]. Additionally, macrophages could eliminate *P. aeruginosa* biofilms in murine model lungs and eradicate biofilms formed in wounds associated to *S. aureus* [217]. A phage lysin has proven to be efficacious in eradicating biofilms by fundamentally cleaving the bacterial cell and also effective towards persister cells. Recombinant exebacase (Lysin CF-301) for treatment of MRSA endocarditis and bacteraemia in synergism with some antibiotics is now in clinical trials [218,219,220]. The stumbling block with macrophage treatment is the resistance formed and their swift clearance by the host immune response. The spatially architectural community of the biofilm allows the dwelling of phage-sensitive bacteria.

### 6.3. Surface Coating or Modification

The multipurpose surface topography and coating on implants are considered as an innovative way to fight against biofilm formation and with the recent emergence in the field of surface engineering new hopes are seen in this arena. The newer techniques like nanoimprint lithography, electron beam and colloidal lithography are utilized for fabrication of textured biomaterial nanosurfaces [221,222]. Coating the implants superficially helps attain the desired outcome without altering the original materials characteristics. Preventing bacterial adhesion, interfering with biofilm formation, and inactivation of the biofilm are the major strategies used in the design of antibacterial coatings. Electrophoretic, chemical vapor and physical vapor deposition are widely used to form uniform thin films on different implants. Recently, AMPs have exhibited inhibition of biofilms and have been used to coat silicon, stainless steel, titanium, glass surfaces, and polystyrene [223,224,225]. Also, antibiotic coating with different classes of antimicrobials like beta-lactam antibiotics, aminoglycosides, rifamycins, and quinolones are explored. Enzymes inhibiting QS are used for coating which includes enzymes such as acylase, oxidase, and lactonase [226,227,228]. Methacrylic copolymer films loaded with a combination of the antibiotics rifampin, clarithromycin and doxycycline efficiently released the drugs for 21 days and prevented formation of biofilms of MRSA, and a combination of clarithromycin with rifampin was able to kill more than 99.9 % of MRSA strains. Combinatory antibiotic therapy provides a good opportunity to bridle the antimicrobial resistance observed with single antibiotics [229,230]. A drawback in the coating strategy is the expeditious eroding of the coating material with time.

### 6.4. Nanoparticle Systems

Owing to the poor permeability concerns coupled with the available drugs towards the persister cells, nanoparticle (NP) delivery systems can be explored for localized drug delivery of antimicrobial agents into biofilms. As evident by several reports, silver nanoparticles (AgNPs) have intrinsic bacteriocidal property and are proven to eradicate biofilms. Siddique et al. studied AgNPs and evaluated their biofilm inhibition against two strains of *K. pneumoniae*. The percent biofilm inhibition was found to be 64% and 86% for *K. pneumoniae* MF953600 and MF953599, respectively at 100 μg/mL concentration [231]. Additionally, the formation of EPS was reduced on treatment with AgNPs and caused cellular membrane disruption. In a similar work by Hetta et al. the biofilm inhibitory activity and antivirulence ability of AgNPs was assessed towards multidrug-resistant *A. baumannii*. The results showed a downregulation in expression of virulence and biofilm-related genes like *afa*/*draBC*, *kpsMII*, *ompA*, *bap*, and *csuA/B* by AgNPs [232]. Recently, Singh et al. studied synthesis of Ag and Au NPs from *Cannabis sativa* and explored their biofilm inhibitory effect towards *P. aeruginosa* and *E. coli* at a concentration range of 1.6–100 µg/mL. AgNPs displayed superior inhibition of biofilm formation even at sub-MIC level [233].

Gounani et al. recently designed tailor-made mesoporous silica NPs as a carrier for the antibiotic vancomycin using surface functionality like amine, carboxyl or aromatic groups. The cellular affinity and attachment with mesoporous silica NP suspensions 0.25 mg/mL was related to a decrease in growth of MRSA biofilm cells [234,235].

A new nanohybrid complex of Ag and iron oxide was designed and subjected to a magnetic field by Sangili et al. These magnetically responsive NPs responded remarkably well compared to bare nanohybrid complexes in disrupting biofilms of *E. coli* and *P. aeruginosa* with inhibitions of 88% and 90%, respectively [236,237]. Abenojar et al. designed a thermoresponsive glycol chitin-based nanocomposite composed of iron oxide nanoparticles and D-amino acids, which transformed from a solution to a gel at physiological temperature with release of D-amino acids. Following the initial disruption by D-amino acids, the nanocomposites combined with thermal treatment were actuated by an external magnetic field to achieve complete disruption of *S. aureus* biofilms [238,239]].

**Figure 14:**
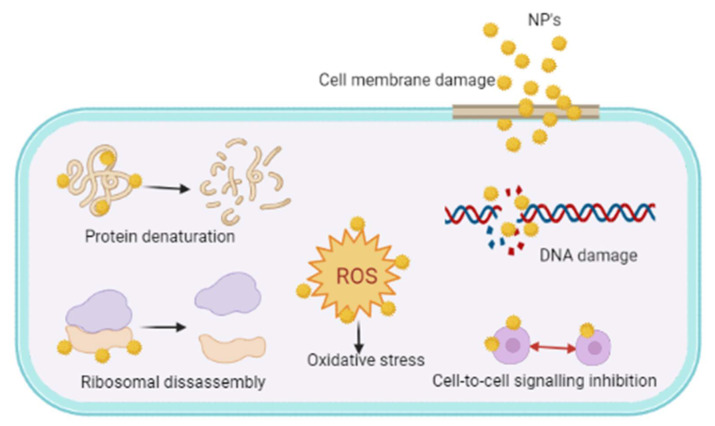
Mechanisms of antibiofilm activities of nanoparticles.

The intricacy in the structure of the biofilms makes the antibiotics difficult to target the bacteria and emergence of resistance to them. Unlike traditional antibiotics, NPs have peculiar dimensions of <100 nm, making them an apt carrier for the antibiotics. These NPs due to their size, help to selectively target the antibiotics to the infestation site thereby lowering the detrimental systemic adverse effects. The NPs exhibit their antimicrobial activity fundamentally by following mechanisms like disrupting the cell membrane and generating ROS. The ROS sequentially initiate intracellular effects by interacting with DNA, ribosomes, enzyme causing oxidative stress associated protein denaturation, DNA damage, ribosomal disassembly and inhibiting cell-to-cell communication within the cell (Figure 14). AgNPs have been shown to induce neutralization of the bacterial membrane surface charges thereby altering its penetrability, eventually leading to cell apoptosis [240,241].

## 7. Conclusions and Future Perspectives

A majority of bacteria found in the environment dwell in the form of sessile biofilms. A careful insight about the complex biofilm biology, its formation process and mode of action aids the design of new antibiofilm agents and strategies. Owing to the hallmark inherent recalcitrance shown by biofilms towards clinically used antimicrobials and the fact that biofilm associated infestations are extremely difficult to treat, there is need for newer antibiofilm agents. Understanding the mode of action of antibiofilm agents provides a roadmap to fill the opportunities in the field. Small-molecule chemical agents hold “drug”-like characteristics and have proven to act against several strains of bacteria resistant to existing antibiotics. QS inhibitors can curb pathogenic infection by blocking cell-to-cell communication and opening up an arsenal for treating biofilm related infections. AMPs have gained interest for their multimodal mechanism of action and reduced the chances of developing resistance. The major pitfall in the use of AMPs is their quick deterioration by several bacterial proteases. QACs have gained notoriety for mimicking the role of the AMPs without undergoing degradation and their inherently small structure comparative to AMPs. The use of naturally derived bioactives have gained immense popularity as biofilm inhibitors owing to their associated safety. Natural phytochemicals in combination with commercial antimicrobials needs to be explored as an alternate strategy to win over the ongoing war against AMR by biofilms. Several novel techniques have been explored for dismantling the biofilm and its related infections.

## Figures and Tables

**Table 1:** Bacterial polysaccharides formed by different organisms and their functions.

Bacterial Polysaccharide	Organisms	Function	References
Polysaccharide intercellular adhesion (PIA)	*Staphylococcus aureus*, *Staphylococcus epidermidis*	Adhesion and architectural framework	[71,72,73]
Pel	*Pseudomonas aeruginosa*	Adhesion, protection and architectural framework	[74,75]
Psl	*Pseudomonas aeruginosa*	Adhesion, protection and architectural framework	[76,77]
Alginate	*Pseudomonas aeruginosa, Pseudomonas syringae*	Protection and architectural framework	[78,79]
Capsular polysaccharides (CPSs)	*Pasteurella multocida, Acinetobacter baumannii, Streptococcus pneumoniae, Vibrio vulnificus*	Protection	[80,81,82,83,84]
Levan	*Pseudomonas syringae, Erwinia amylovora, Bacillus subtilis, Streptococcus mutans*	Adhesion and protection	[85,86]
Colanic Acid	*Enterobacteriaceae*	Architectural framework	[87,88]
Vibrio	*Vibrio cholerae*	Adhesion, architectural framework	[89,90,91,92]
α-Mannans and β-glucans	*Candida albicans*	Forming mannan-glucan complex (MGC) and protection	[93,94]
Glucans/fructans	*Streptococcus mutans, Weissella cibaria, Lactobacillus plantarum*	Adhesion and protection	[95,96,97]

**Table 2:** BIC50 values for 1,2,4-oxadiazole topsentin analogues.

Compound	R	R^’^	BIC50 Values for *S. aureus* (μM)
(5-floro-1-methyl-1H-indol-3-yl)[3-(5-bromo-1-methyl-1H-indol-3-yl)-1,2,4-oxadiazol-5-yl]methanone (**5a**)	F	Br	4.4
(5-floro-1-methyl-1H-indol-3-yl)[3-(5-floro-1-methyl-1H-indol-3-yl)-1,2,4-oxadiazol-5-yl]methanone (**5b**)	F	F	0.27
(5-methoxy-1-methyl-1H-indol-3-yl)[3-(5-methoxy-1-methyl-1H-indol-3-yl)-1,2,4-oxadiazol-5-yl]methanone (**5c**)	OCH_3_	OCH_3_	0.9
5-fluoro-1-(2-methoxyethyl)-3-[4-(thiophen-3-yl)-1,3-thiazol-2-yl]-1H-indole (**6a**)	-	-	3.9
5-bromo-1-(2-methoxyethyl)-3-[4-(pyridin-3-yl)-1,3-thiazol-2-yl]-1H-indole (**6b**)	-	-	1.0

**Table 3:** IC50 values for amide chalcones.

Compound	R	Bacterial Biofilm Inhibition (IC50 in μM ± SD)		
		*S. aureus*	*M. luteus*	*P. aeuroginosa*
(E)-N-(4-(3-(4-Chlorophenyl)acryloyl)phenyl)-3-(piperidin-1-yl)propanamide (**7a**)	4-Cl	2.4 ± 0.10	4.8 ± 0.11	7.8 ± 0.24
(E)-N-(4-(3-(4-Methoxyphenyl)acryloyl)phenyl)-3-(piperidin-1-yl)propanamide (**7b**)	4-OCH_3_	4.9 ± 0.21	5.7 ± 0.26	8.6 ± 0.22
(E)-3-(Piperidin-1-yl)-N-(4-(3-(3,4,5-trimethoxyphenyl)acryloyl)phenyl)propanamide (**7c**)	3,4-di(OCH_3_)	2.9 ± 0.16	5.6 ± 0.22	0.84 ± 0.21

**Table 4:** Biofilm inhibition ratios for cajaninstilbene acid derivatives.

Compound	R	Biofilm Inhibition Ratio (%)
2-hydroxy-4-methoxy-3-(3-methylbut-2-en-1-yl)-6-[(Z)-2-(pyrimidin-5-yl)ethenyl]benzoic acid (**8a**)	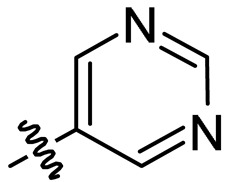	36.11 ± 0.58
2-hydroxy-4-methoxy-3-(3-methylbut-2-en-1-yl)-6-[(Z)-2-(quinolen-8-yl)ethenyl]benzoic acid (**8b**)	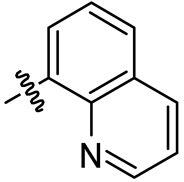	35.61 ± 3.76
2-hydroxy-4-methoxy-3-(3-methylbut-2-en-1-yl)-6-[(Z)-2-(sulfurpentafloro-benzene-4-yl)ethenyl]benzoic acid (**8c**)	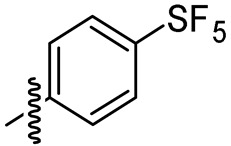	49.50 ± 1.35

## Data Availability

Not applicable.
